# Loss-of-function genetic diseases and the concept of pharmaceutical targets

**DOI:** 10.1186/1750-1172-2-30

**Published:** 2007-07-02

**Authors:** Laurent Ségalat

**Affiliations:** 1 CGMC, CNRS-UMR 5534, Université Lyon 1, France

## Abstract

The biomedical world relies heavily on the definition of pharmaceutical targets as an essential step in the drug design process. It is therefore tempting to apply this model to genetic diseases as well. However, whereas the model applies well to gain-of-function genetic diseases, it is less suited to most loss-of-function genetic diseases. Most common diseases, as well as gain-of-function genetic diseases, are characterized by the activation of specific pathways or the ectopic activity of proteins, which make well identified targets. By contrast, loss-of-function genetic diseases are caused by the impairment of one protein, with potentially distributed consequences. For such diseases, the definition of a pharmaceutical target is less precise, and the identification of pharmaceutically-relevant targets may be difficult. This critical but largely ignored aspect of loss-of-function genetic diseases should be taken into consideration to avoid the commitment of resources to inappropriate strategies in the search for treatments.

## Target identification in the drug discovery process

**Figure 1:**
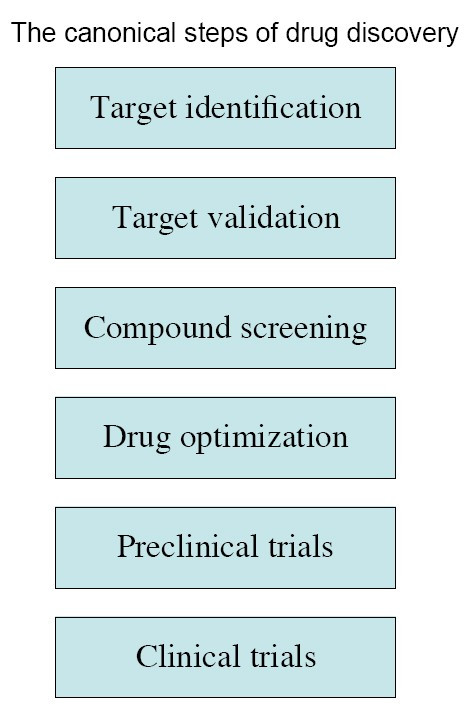
**The canonical steps of modern drug discovery**. Target identification is the first and obligatory step in the canonical paradigm of drug discovery.

Target identification is an essential first step in today's drug discovery process. This principle is built on the premises that, to be efficient, drug development must be directed against identified biological mechanisms at molecular level (pharmaceutical targets). This concept is the core of modern drug discovery and is the primary difference between modern and former drug discovery, when drugs were discovered more empirically than rationally, and were put on the market without much knowledge of their targets. Today's drug discovery has, by contrast, become largely target-dependent [1]. The industry standard for drug discovery is defined by an almost invariant frame consisting of sequential steps: target identification, target validation, compound screening/design, compound optimization, preclinical (animal) trials, and clinical trials (Figure 1) [2,3].

This conception of the drug discovery process is commonly accepted beyond the industrial world and is deeply rooted into the biomedical field, including academia. The sequencing of large genomes is often justified as a way to enlarge the repertoire of targets [2]. The sequencing of the human genome, for instance, was sold as such to the decision-makers. The pharmaceutical industry's appetite for targets is filled by academic laboratories and by spin-off companies specialized in the commercial identification of targets. Although the pharmaceutical industry has experienced a decline in bringing new drugs to the market in recent years, the target-based approach remains by far the dominating drug discovery paradigm [3,4].

Since rare diseases were neglected for decades by both policy-makers and industry, drug discovery for rare diseases has a relatively short history, and remains a dwarf in terms of expenditure when compared to drug discovery for common diseases (cardiovascular diseases, cancer, diabetes, Alzheimer's disease, *etc*.). It may, therefore, sound like a reasonable endeavor to apply to an emerging field a strategy that is the standard in another not-so-distant field. For this reason, when it comes to genetic diseases, a shared view exists within the scientific community that the target-based drug development concept should apply to genetic diseases as it does to non-heritable diseases. This one-size-fits-all strategy is conservative but, as we will see, may not be the optimal strategy, since some genetic diseases are only poorly adapted to it.

## Genetic diseases fitting the concept of a therapeutic target

### Gain-of-function diseases

Gain-of-function (gof) genetic diseases, as the term indicates, are caused by the ectopic or increased activity of the mutated gene product. Proteins mutated in gof diseases may or may not carry a dominant-negative effect. Ectopic or increased protein activity most often turns on cellular processes that normally do not occur in a healthy cell, thereby triggering a pathology. In this respect, gain-of-function genetic diseases are comparable to the common diseases that are in the focus of the pharmaceutical industry (cancer, stroke, infectious diseases, Alzheimer's disease) and that may also be defined, in first approximation, as an activation of pathological cellular processes that do not occur in a healthy cell. Therefore, it is of no surprise that the concept of the target-based drug design applies well to this class of diseases. Who could claim to have a better approach against polyglutamine expansion diseases, for instance, than neutralizing the faulty protein and blocking the downstream chain of deleterious events?

### Loss-of-function diseases

**Figure 2:**
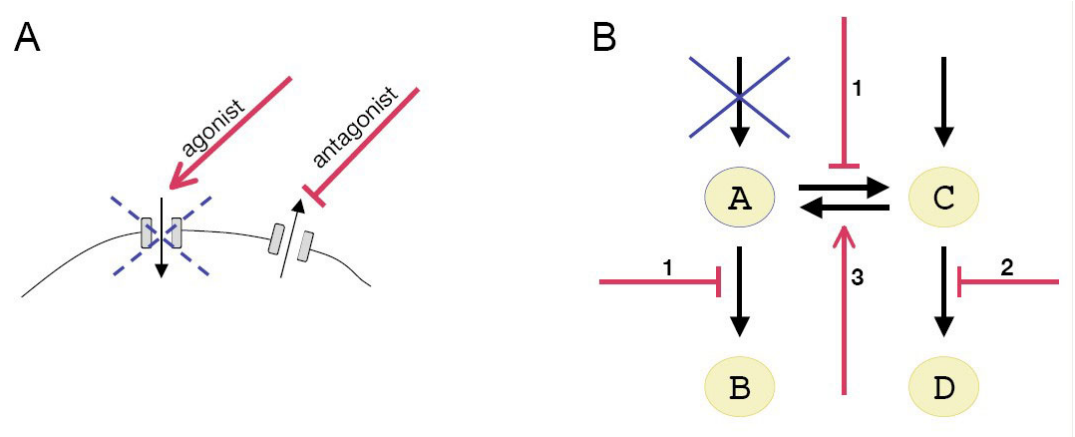
**Only a few specific loss-of-function genetic diseases match the concept of a pharmaceutical target**. Although the concept of a pharmaceutical target does not fit most loss-of-function diseases, channelopathies and some metabolic diseases are exceptions to the rule. A) Schematic view of putative strategies to treat a partial loss-of-function affecting an ion channel. Agonists may stimulate the channel to increase its activity. Alternatively, antagonists of opposite-effect channels may restore the ion balance of the cell. B) Schematic view of putative strategies to treat a loss-of-function disease affecting an enzyme. In this example, the reduction of enzyme activity (blue cross) results in a deficit in a key metabolite A. This deficit may be compensated by inhibiting A-transforming enzymes (1), increasing the abundance of a precursor (2), and stimulating A-producing enzymes (3). (Supplementation of A, also a therapeutic possibility for some disorders, is not shown.)

A small proportion of loss-of-function (lof) genetic diseases are also well-suited to the concept of target-based drug design. These are mostly diseases with a simple and well-understood physiopathology, like channelopathies and some metabolic diseases. In such diseases, the reduction of a cellular function resulting from the mutation may be pharmacologically corrected by either stimulating this function or inhibiting an opposite function (Figure 2). For instance, in Myotonia congenita (a recessive disease caused by mutations in the chloride channel gene *CLCN1*), malfunction of the chloride channel impairs the skeletal muscle repolarization, the voltage-dependent sodium channels are improperly opened, and a subsequent myotonia ensues. Myotonia symptoms can be effectively reduced by a sodium channel antagonist, mexiletine, which decreases the muscle excitability, and thereby re-establishes the balance between excitation and relaxation [5] (Figure 2A). The concept of pharmaceutical targets (chloride channel and sodium channel in this case) makes perfect sense here because i) the physiology of muscle excitation is well-known and ii) channels are highly amenable to modulation by drugs.

Another example is provided by metabolic diseases. Metabolic diseases are a physiologically-damaging alteration in the amount of a key cellular metabolite, and are often caused by loss-of-function mutations in enzyme-encoding genes. When supplementation treatments are not possible, metabolic diseases may be treated by pharmacological strategies aimed at restoring an appropriate level of the key metabolite by playing with adjacent metabolic pathways (Figure 2B). Here again, the relatively simple physiopathology allows for an easy identification of therapeutic targets.

## Genetic diseases for which the concept of a pharmaceutical target is, at best, vague

**Figure 3:**
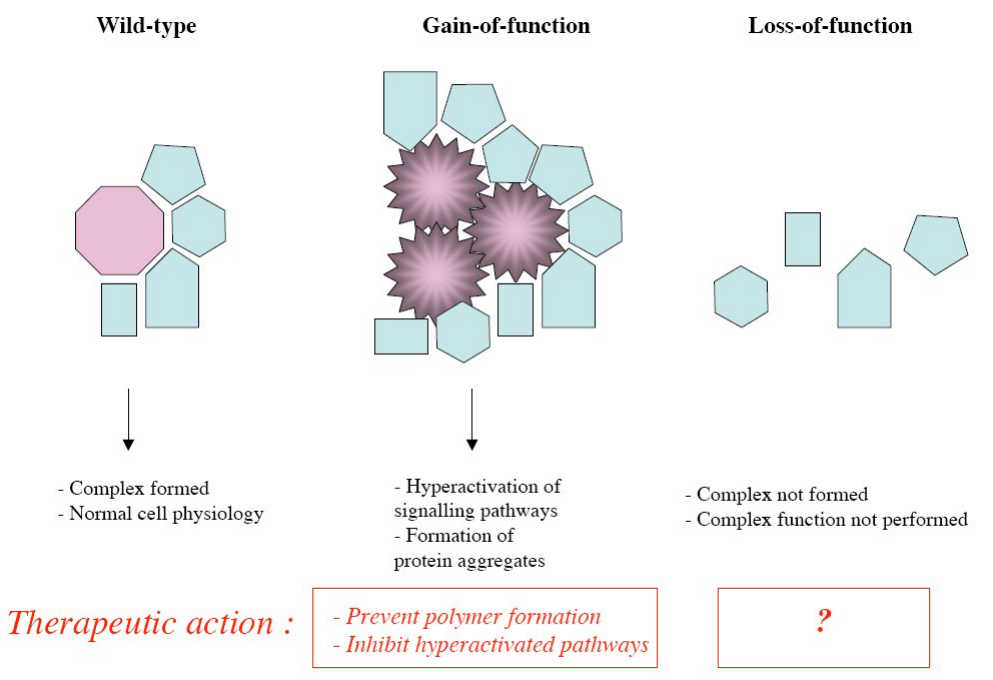
**Schematic view of the physiological consequences and possible countermeasures against gain-of-function and loss-of-function mutations**. In this example, the protein of interest (pink octogon) normally supports the assembly of a protein complex. (LEFT) In the wild-type situation, a complex is formed with other protein partners (blue polygons). (MIDDLE) A gain-of-function mutation results in the abnormal formation of protein polymers and protein aggregates, which may have constitutive activity or be toxic to the cell. Therapeutic options will consist in neutralizing the mutated protein and blocking the formation of aggregates with other proteins; pharmaceutical targets are already identified or may be identified readily in downstream pathways. (RIGHT) A loss-of-function mutation will result in the absence of the complex. The biological processes depending on the complex will be perturbed. Pharmaceutical targets are more evasive in the loss-of-function situation.

Unfortunately, the concept of a pharmaceutical target fits poorly with the majority of loss-of-function genetic diseases. This is due to the specific position that loss-of-function genetic diseases occupy in the spectrum of disorders: it is conceptually difficult to correct the absence of a protein by pharmacological means (Figure 3). This simple but largely overlooked fact has broad implications for drug discovery.

Duchenne muscular dystrophy (DMD), one of the most studied genetic diseases, is due to the impairment of dystrophin, a structural protein underlying the muscle membrane. After 20 years of research on dystrophin, neither the role of dystrophin in a healthy muscle nor the physiopathology of the disease are fully understood yet [6,7]. It is established that the absence of a functional dystrophin results in muscle fibre calcium overload, mislocalization of signaling proteins, and membrane fragility. Calcium overload may in turn translate into dozens of secondary effects, ranging from increased muscle excitability to improper caspase activation. Mislocalization of signaling proteins and membrane fragility, the two other main traits of DMD, may also be subdivided into numerous items, some of them being deleterious and others not. What, then, causes the end-point phenotype of progressive muscle necrosis? Is it just one of the many secondary effectors or, more likely, a combination of several of them? As in many other diseases affecting structural proteins, the loss-of-function mutation has a pleiotropic effect, distributed amongst various cellular functions, pathways and compartments.

Pleiotropy is also a hallmark of diseases affecting genes involved in central cellular processes: DNA replication and maintenance, transcription and RNA processing, protein maturation, trafficking, *etc*. Spinal muscular atrophy type I (SMA 1), one of the most frequent genetic diseases, is caused by mutations in the *SMN *gene. Although the genetics of the *SMN *locus is complex, it can be considered as a lof disease, since it results in a reduction of functional SMN protein. The SMN protein plays a role in spliceosomal snRNP biogenesis, and *SMN *mutations seem to result in a shortage of functional spliceosomes [8,9], thereby globally perturbing mRNA processing. The number of misprocessed mRNA species is not yet fully established, but is probably very large. How many of them contribute to the progressive death of motorneurons? Probably more than just a few.

As with the mRNA processing genes, so too with the transcription factor genes. How many misregulated genes are responsible for the numerous symptoms observed in Cleidocranial dysplasia (CCD) and Waardenburg syndrome, to mention only these two diseases among the many affecting transcription factors?

What are the pharmaceutical targets for Emery-Dreifuss dystrophy (mutation in a nuclear envelope protein), for Schwartz-Jampel disease (mutation in the basement membrane protein perlecan), for Centronuclear congenital myopathy (mutation in dynamin, a transport vesicle protein)?

These few examples demonstrate that the concept of a pharmaceutical target – a biochemical entity which can be levered by a drug – is unsuitable to many loss-of-function genetic diseases.

## Risks and difficulties of applying the «target-first» strategy to physiologically complex genetic diseases

Twenty years ago, in the early days of human molecular genetics, the scientific community propagated the erroneous idea that, once a disease gene was identified, the treatment would be around the corner. The following years have been sobering and disillusioning. We should be careful not to repeat that mistake by spreading another idea, which might be as over-optimistic as the previous one: that genetic diseases will be modeled around «targets», and that targets will be the key to treatments. In this view, targets identified by biologists will be passed on to medicinal chemists who will design target-based treatments. This scheme is in line with the contemporary approach of drug discovery. Unfortunately, when it comes to genetic diseases, two factors greatly reduce the feasibility of this strategy: i) the definition of pharmaceutical targets is, at best, vague, for the majority of lof genetic diseases; ii) to have some chance of success, a target-designed treatment against the above-mentioned diseases should be directed against not one but several targets simultaneously – an almost impossible challenge.

There is another contextual element which is rarely mentionned, but which should also be looked at with open eyes. The contemporary drug discovery strategy has scored some spectacular successes on certain fronts. However, other less successful stories remind us that it is by no means a trivial matter to battle diseases, even when the targets are clearly identified and the picture looks simple. In the fight against infectious diseases, for instance, in which the pathological process starts by a sequential and almost linear chain of well-characterized events, we are still a long way from total victory. The same is true for cancer. Some forms of cancer are far from contained, despite well-known targets and big spending. What then, are the chances of finding treatments against genetic diseases on a large scale, by applying the same and, in this case, a less appropriate strategy with much less money?

## What should be done?

### 1. Invest in the understanding of the physiopathology, but not for the reason usually put forward

This dark picture does not mean that money invested in trying to dissect out the mechanisms underlying physiologically complex lof genetic diseases is useless. It is important to carry on investing in the understanding of the physiopathology of these diseases, but the main reason to do it might not be the one usually put forward (finding targets that pharmacologists and chemists will turn into treatments). Otherwise, the scientific community may once again promise more that it can deliver.

The first reason for investing in the understanding of genetic disease physiopathology is that even though the efficiency of the «target first» strategy as a global approach has been overestimated, its fallout on specific diseases must not be neglected. There will be instances when findings involving the physiopathology will shed light on mechanisms rapidly amenable to drug therapies, such as idebenone against Friedreich ataxia [10]. However, such situations are rare and there is no reason to think that their proportion will increase.

**Figure 4:**
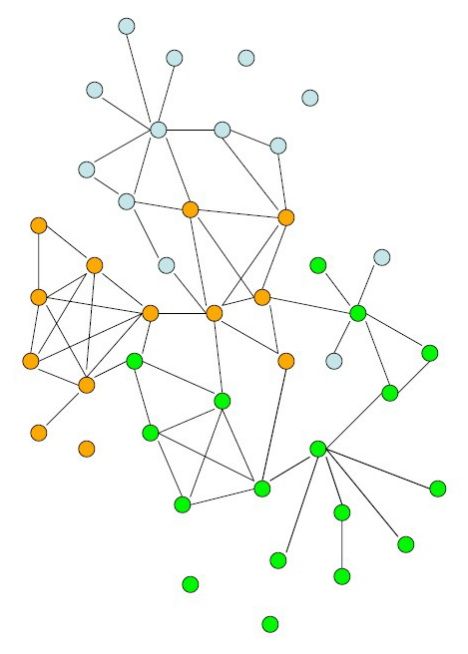
**Thinking global in the fight against genetic diseases**. Genetic diseases (circles) can be put on virtual maps based on multiple levels of biological information. Once treatments are available for a few diseases, taking advantage of the connections will facilitate the identification of treatments for other diseases.

The main drive to invest in the understanding and the fine characterization of genetic disease physiopathology is that it creates a knowledge environment. This knowledge environment has at least two benefits: Firstly, it will be an essential tool to evaluate the efficiency and the mode of action of future treatments, which may or may not (see below) originate from a target-based approach. Increasing the number of available parameters and endpoints allows for a better appraisal of treatment efficiency. Secondly, with 5,000 or more genetic diseases around, one *has*to think global. The knowledge environment developed on each disease (or group of diseases) can be used to chain diseases togetherin a network of information. Genetic diseases, even those for which little is known, can be placed on interactome-like virtual maps (Figure 4) based on all sorts of information, ranging from literature keywords to transcriptome gene clustering and bioprofiling of body fluids. As treatments appear for some diseases, taking advantage of the network connections will rapidly provide therapeutic hints for other still-orphan diseases, and may constitute the largest payoff of the investment made on trying to dissect the physiopathology.

### 2. Consider alternative strategies

It is probably time to reevaluate the choice of the «target first» strategy as the main option in the search of pharmaceutical treatments against physiologically complex lof genetic diseases. For these diseases, success may well come from a less ambitious but more pragmatic strategy: the «screen first, understand later» strategy. This alternative strategy, also referred to as physiology-based, is of interest in all indications where no obvious target is available [7]. In the search for treatments against rare diseases, the bonanza of existing drugs has been surprisingly underexploited up to now. Yet, anecdotal evidence demonstrates that old drugs can have an unpredicted beneficial effect on some genetic diseases: colchicine was serendipitously found to cure Familial mediterranean fever (FMF), and acetazolamide is active against Episodic ataxia despite any rationale underlyings. Since these discoveries are the result of sheer luck (co-occurrence of diseases in one case and diagnostic error in the other), one can extrapolate that the pharmacopea contains many more unraveled gems. Furthermore, beyond approved drugs, chemical libraries constitute another vast reservoir of potentially useful molecules against genetic diseases. These chemical resources should be taken advantage of more thoroughly. Currently, the limiting factor for molecule identification is the shortage of high-throughput screening (HTS) systems. For most genetic diseases, *in vitro *models either do not exist or are not suitable for HTS screening. This bottleneck is less a technological one than the result of insufficient development. Committing financial resources to overcome this limitation and running high-throughput molecule screens might at the end of the day be one of the most cost-efficient investments in rare disease research.

## Conclusion

After being ignored by governments for decades, rare diseases are now a biomedical research priority in most developed countries. However, the fundamental difference that exists between loss-of-function genetic diseases (the most numerous ones) and most other human disorders has been largely overlooked. As a consequence, confusion exists in the definition of what the optimal drug-discovery strategy against the majority of loss-of-function diseases is. In-depth reflection is necessary to resolve this issue and to avoid the commitment of energy and money to inappropriate research strategies.
